# *Plantago* species are emerging model organisms for functional genomics and stress biology

**DOI:** 10.1007/s00299-025-03530-w

**Published:** 2025-06-17

**Authors:** Hannah Levengood, Lillian Smith, Shelby Gillis, Yun Zhou, Cankui Zhang

**Affiliations:** 1 Department of Agronomy, Center for Plant Biology, Purdue University, West Lafayette, IN 47907 USA; 2 Department of Botany and Plant Pathology, Center for Plant Biology, Purdue University, West Lafayette, IN 47907 USA

**Keywords:** *Plantago*, Model organisms, Vascular biology, Ecology, Medicinal plant

## Abstract

**Key message:**

**Species in the Plantago genus are emerging model organisms to multiple research disciplines.**

**Abstract:**

The genus *Plantago* has long been recognized for its significance in various research fields, yet it remains underutilized as a model organism in scientific studies. Several *Plantago* species possess unique traits, including easily accessible vascular tissues, medicinal properties, gynodieocity, and remarkable adaptability to diverse environmental conditions. These characteristics position *Plantago* as a promising model for research in areas such as plant vascular biology, stress physiology, reproductive biology, ecology, and medicinal biochemistry. Recent advancements, including the development of genetic transformation systems, the availability of sequenced genomes, and the application of CRISPR-Cas9 technology, have significantly enhanced the capability of using *Plantago* as a model system. This review discusses the research potential of *Plantago* species, highlighting key historical discoveries and recent breakthroughs that demonstrate their value across multiple scientific disciplines*.*

## Introduction

The use of plant model systems to study key aspects of plant biology is widely recognized as essential. Plant models are typically desirable for scientific studies because they offer extensive genomic resources and traits which make them easily adapted to a laboratory environment. Among these, Arabidopsis is the most commonly used model in plant biology, due to its advantageous features such as a short life cycle, broad geographical distribution, a reliable plant transformation system, and a well-characterized, sequenced genome. These attributes have enabled its widespread adaptation across various scientific fields. However, Arabidopsis and other commonly used plant models (e.g. petunia, tobacco, tomato) have limitations that restrict their ability to address certain biological questions (Cowley and Burton 2021). Therefore, promoting the development and adoption of new plant models is crucial for advancing scientific research.

While many plants, such as corn and soybean, have been used as experimental organisms, their results often remain specific to their genus or research area. In contrast, new plant model species should have broader applicability, enabling discoveries that advance plant biology across multiple fields (Bertile et al. 2023). Like Arabidopsis, ideal models should possess small genomes for ease of sequencing, be easily manipulated in laboratory settings, and have a reproducible system for genetic manipulation. Additionally, these species should offer unique traits that make them particularly advantageous for certain types of studies, thereby increasing their value in scientific research.

Recently, several species have been proposed as alternative models to Arabidopsis, including duckweed (*Lemnaceae)* (Zhang et al. 2010)*, Physcomitrium patens* (Liu and Vidali 2011)*, Marchantia polymorpha* (Community 2024)*,* and *Plantago* (Levengood et al 2024). The *Plantago* genus, in addition to having small genomes and published transformation systems, has a long-standing history as a focal group in diverse research areas. These include, but are not limited to, medicine, ecology, floral symmetry and cell wall formation. Species in the genus also exhibit unique features like male sterile flowers and easily removable vascular tissue, which have facilitated significant discoveries in floral biology and plant vascular biology.

In addition to serving as valuable models for scientific study, the *Plantago* genus has been significant to human culture for thousands of years. Historically, *Plantago* pollen grains have been used in anthropological studies as evidence of human settlements, with human use dating back to the early Neolithic period (Rösch and Lechterbeck 2016). The *Plantago* genus originated in Eurasia and then radiated and specified to the rest of the world, and thus have been widely used in Europe, Asia, and later in the Americas. They are referenced in ancient texts such as *De Materia Medica* for their wound healing properties and were recommended by Pliny the elder for treatment of diseases (Weryszko-Chmielewska et al. 2012). These plants also appear in Anglo-Saxon charms (Stride 2017) and Shakespearean works. In Asia, *Plantago* occupies an important place in Chinese and Persian medicinal traditions for its healing properties (Nelson 2016). Today, the uses of *Plantago* extend beyond traditional medicine to nutrition and commercial products. *Plantago* leaves are consumed globally in salads, soups, and teas, and are even incorporated into animal feed to enhance health and meat quality (Weryszko-Chmielewska et al. 2012). Extracts from these plants are also used in cosmetics for their antiseptic and astringent properties.

This review highlights the diverse applications of *Plantago* species as research models across various scientific disciplines. We emphasize both recent breakthroughs and significant historical contributions achieved through studies on *Plantago*. Additionally, we discuss recent efforts aimed at advancing *Plantago* as a prominent model organism for future research endeavors.

## Characteristics

**Fig. 1:**
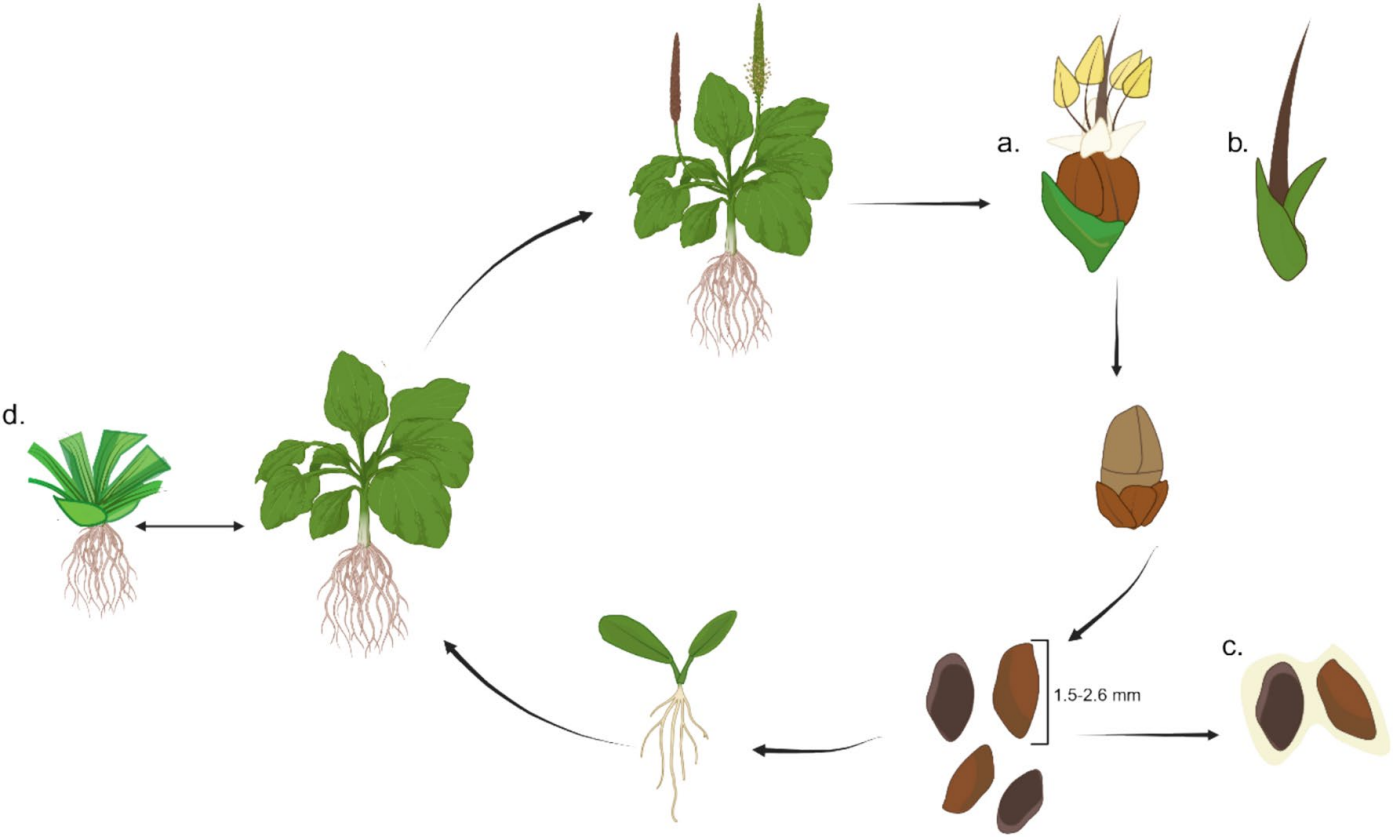
Generalized life cycle of *Plantago* species. Flowers can be hermaphrodites (**a**) or male sterile (**b**), depending on the species. Seeds are generally small, 1.5–2.6 mm, and can produce a gelatinous mucilage when wet to aid dispersal (**c**). Species can be annual or perennial, and those that overwinter lose their upper vegetation, and can regenerate from crowns each spring (**d**)

The approximately 250 species that comprise the *Plantago* genus are generally small, rosette forming herbs (Penczykowski and Sieg 2021). Many plants in the genus are characterized by a short stem, about 1 cm in length, from which the rosette and roots emerge (Sagar and Harper 1964). Many morphological traits, such as rosette form, floral shape and inflorescence structure, venation and root morphology, are consistent among the species (Klimeš 1995). However, exceptions do exist, such as the male sterile phenomenon observed in *P. lanceolata* (Fig. 1b). Other characteristics are influenced by selection pressure, resulting in specialized traits in some species. For example, *P. maritima,* which inhabits coastal areas, exhibits remarkable salt tolerance (Hediye Sekmen et al. 2007). Depending on the species, *Plantago* can be either diploid or polyploid (Penczykowski and Sieg 2021).

In general, most species in the *Plantago* genus have relatively short life cycles. *P. major* plants in the wild typically flower 6–10 weeks after emergence (Sagar and Harper 1964), although shorter timelines have been observed under laboratory and greenhouse conditions (Levengood et al. 2023). A representative life cycle of *Plantago* species is shown in Fig. 1. The flowers are generally hermaphroditic (Fig. 1a), wind-pollinated or self-fertile, although gynodioecious plants do exist in the genus. For example, *P. lanceolata* exhibits both hermaphroditic and female (male-sterile) flowers (Fig. 1b) (Nugent et al. 2019). The number of seeds produced varies depending on environmental conditions but are generally produced in large amounts. *P. major* can produce up to 14,000 seeds per plant annually, while *P. lanceolata* can yield up to 10,000 (Cavers et al. 1980). Seed size and shape differ among species, with many producing gelatinous mucilage (Fig.1c) when wet. Mucilage is an important aspect of *Plantago* biology, having versatile functions such as seed dispersal, defense, germination, and root elongation (Teixeira et al. 2020a, b). Perennial species in the genus typically lose their upper vegetation in the winter, although they can regenerate from crowns in the spring (Fig. 1d).

**Fig. 2:**
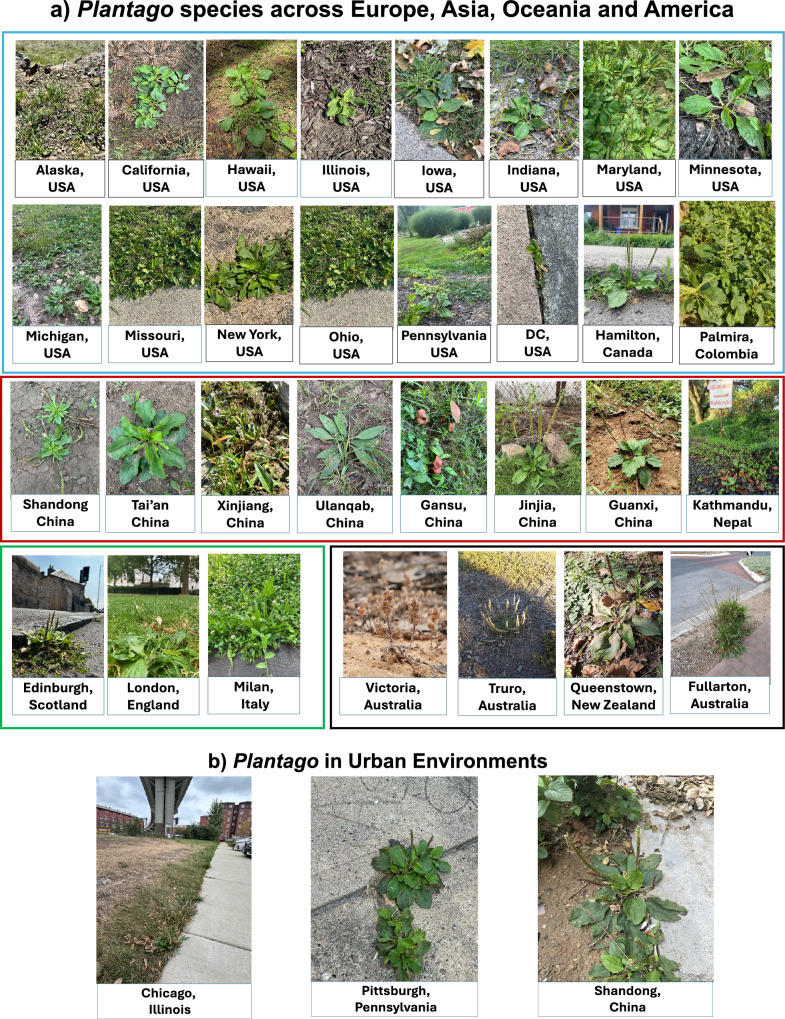
Plantago species have a wide geographic distribution. **a** Blue frame represents selected *Plantago* species identified in various states of the United States of America, Canada and Colombia. Red frame represents selected *Plantago* species identified in various regions of Asia. Green frame represents selected *Plantago* species identified in Europe. Black frame represents selected Plantago species identified in Oceania; Australia and New Zealand. **b** Examples of *Plantago* in urban areas. Names of towns and countries/states where the pictures were taken are documented, as applicable

*Plantago* species exhibit remarkable adaptability, enabling them to thrive across diverse environments and attain a widespread geographic distribution. They are present on most continents, except for Antarctica, and can be found in nearly all states of the United States, including Alaska and Hawaii, which demonstrates their exceptional tolerance to varying climatic conditions. Similarly, *Plantago* species are prevalent throughout Europe, Oceania, China, and other parts of Asia, as well as most temperate and tropical regions globally (Klimeš 1995). Although most species historically appear alongside agriculture due to landscape disturbances, some species, like *P. major,* are also cosmopolitan in their distribution and have also thrived in urban environments. Figure 2 illustrates examples of *Plantago* species captured throughout the world. Additionally, urban examples of *Plantago* are also highlighted to show their ability to thrive in anthropogenic landscapes.

## Breakthroughs and discoveries using *Plantago*

Currently, a number of species in the *Plantago* genus have been used as experimental models across a wide range of research areas. To identify which species in the genus are most studied, and quantify their representation in scientific literature, we performed literature searches on the NCBI PubMed database. On October 24th, 2024, we queried the database using the search terms: “Plantago [species name]” for all species within the *Plantago* genus. Species were ranked by their total number of records on Web of Science, with the ten most studied species reported in Table 1. *P. lanceolata* emerged as the most frequently studied species, with 5563 records on Web of Science, followed by *P. major* (2782), *P. ovata* (1924), and *P. coronopus* (869). Interestingly, each of the ten most studied species have been utilized to advance research in multiple fields, with Ecology, Agriculture, and Genetics being the most frequently studied fields that use *Plantago*. This is another testament to the versatility of *Plantago* as a model system. Several significant discoveries and breakthroughs achieved using *Plantago* in these areas are summarized below.

**Table 1:** 10 Most studied Plantago species

Species	Common name	Research articles—web of science	10 most common research areas (by published papers)
*P. lanceolata*	Ribwort plantain, Narrowleaf plantain, English plantain, Buckhorn plantain	5563	Plant science, ecology, agriculture, conservation, physiology, biochemistry/molecular biology, nutrition, dietetics, chemistry, genetics
*P. major*	Broadleaf plantain, Greater plantain, Common plantain, White-man’s footprint	3782	Plant science, agriculture, ecology, pharmacology, biochemistry, chemistry, physiology, conservation, pathology, genetics
*P. ovata/* *psyllium*	Blond plantain, Desert Indianwheat, Blond psyllium, Ispaghol	1924	Plant science, agriculture, pharmacology, chemistry, food science, environmental science, biochemistry, nutrition, genetics, physiology
*P. coronopus*	Buck’s horn plantain	867	Plant sciences, ecology, agriculture, biomedicine, conservation, genetics, physiology, reproductive biology, anatomy, evolutionary biology
*P. media*	Common plantain, Hoary plantain, Waybread	784	Plant science, ecology, agriculture, conservation, physiology, biochemistry, biomedicine, genetics, anatomy, cell biology
*P. maritima*	Sea plantain, Seaside plantain, Goose tongue	747	Ecology, plant science, conservation, agriculture, physiology, biomedicine, marine freshwater biology, genetics, developmental biology, anatomy
*P. arenaria*	Branched plantain, Sand plantain, Black psyllium	524	Plant sciences, agriculture, ecology, pharmacology, chemistry, nutrition, dietetics, physiology, biochemistry, life sciences
*P. patagonica*	Woolly plantain	135	Ecology, plant sciences, agriculture, conservation, genetics, physiology, biochemistry, reproductive biology, cell biology, evolutionary biology
*P. lagopus*	Rabbit’s foot plantain, Lagopus plantain, Hare’s foot plantain	127	Plant sciences, genetics, environmental sciences, cell biology, agriculture, biochemistry, biomedicine, anatomy, chemistry, evolutionary biology
*P. rugelii*	American plantain, Blackseed plantain, Pale plantain, Rugel’s plantain	99	Plant science, ecology, agriculture, biomedicine, physiology, biochemistry, conservation, evolutionary biology, genetics

## Vascular biology

Vascular tissue, i.e., xylem and phloem, play a key role in various biological processes, making them a significant focus for plant biological research. These tissues are responsible for both local and long-distance nutrient transport. As a result, numerous genes and pathways associated with nutrient transport and responses to nutrient deficiency have been discovered through studies of plant vasculature. Plant vascular tissue is also important for systemic signaling associated with plant development and stress biology. These include systemic acquired resistance (SAR) to pathogens (Al Mamun et al. 2024), systemic wound responses (SWR) (Wu et al. 2024), adaptation to low mineral growth conditions (Xia et al. 2020; Smith et al. 2022; Ding et al. 2020), and root development (Liu et al. 2024a, b).

To accurately study genes and molecular pathways associated with systemic signaling, it is essential to obtain pure vascular tissue. However, this has been challenging due to the complexities associated with precise tissue collection. Currently, three primary methods are utilized for phloem sampling: EDTA-facilitated sampling, stylectomy, and cucurbit bleeding. These methods, each with their own advantages and limitations, are described below.

(1) EDTA-facilitated sampling is the most widely used approach to collect phloem sap. In this method, petioles are excised, and the cut ends of the petioles are submerged in a buffer containing EDTA. The EDTA chelates Ca^2^⁺ ions and prevents the plant’s wound-healing response, resulting in exudating of the phloem sap (Xu et al. 2019). However, concerns about contaminations from damaged tissue caused by EDTA have been raised (Liu et al. 2012). (2) Stylectomy is another method in which severed stylets of phloem-feeding insects are used to collect phloem sap. While this technique allows precise sampling, it is technically challenging, requiring the severing of stylets and sap collection to be performed under a microscope. Additionally, the extended duration of the procedure can induce secondary effects, potentially altering the molecular processes under study. (3) Curcurbit plants have been used in some studies to investigate phloem physiology due to their unique ‘bleeding’ characteristic. However, some signals produced in the phloem companion cells do not enter the phloem sap and cannot be identified using this method (Zhang et al. 2012).

In addition to these traditional methods, a few advanced tissue-specific collection techniques can be employed to investigate phloem specific molecular responses. These include laser capture micro-dissection (LCM) (Ludwig and Hochholdinger 2014), fluorescence-activated cell sorting (FACS) (Galbraith et al. 1998), and translating ribosome affinity purification (TRAP) (Mustroph et al. 2009). LCM of phloem cells is technically challenging and requires special instruments. TRAP and FACS are in theory appropriate methods to study phloem specific responses, but both approaches require the development of transgenic plants (Galbraith et al. 1998; Mustroph et al. 2009).

**Fig. 3:**
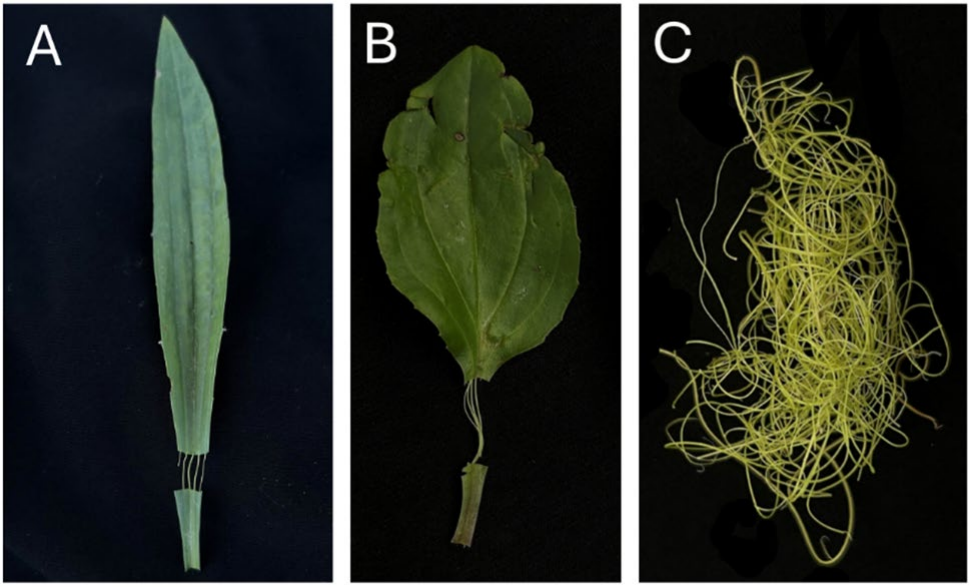
Vascular tissue can be easily obtained from various species in the *Plantago* genus. **A** *Plantago lanceolata*. **B*** Plantago major*. **C** Vascular tissues extracted within few minutes

**Fig. 4:**
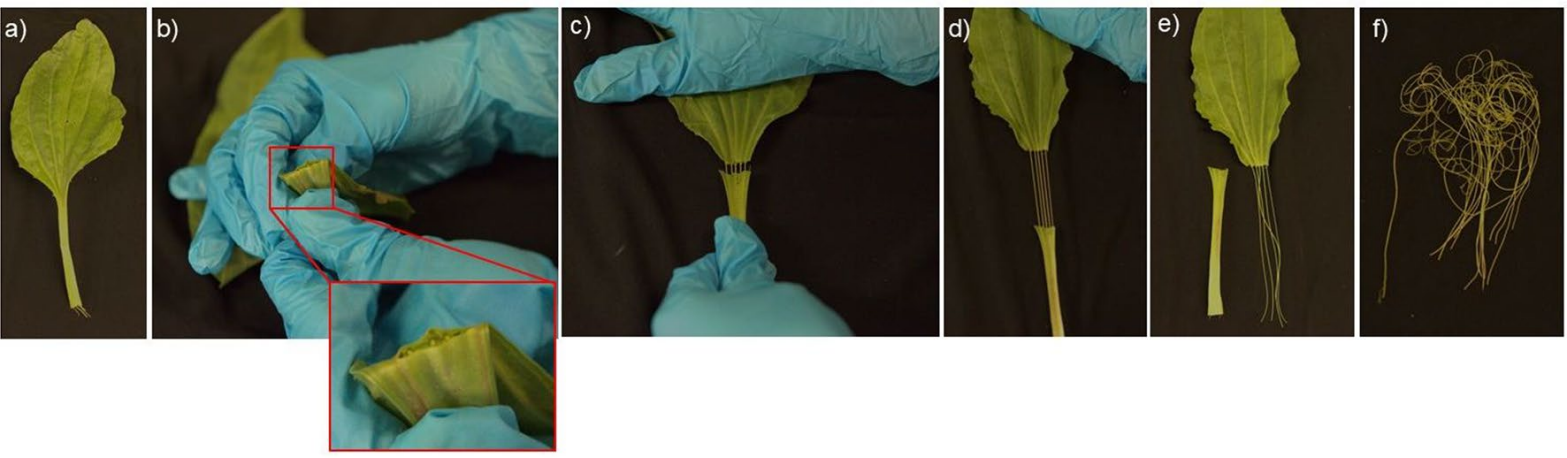
Vascular tissue from *Plantago* is easy to extract. **a** Intact *Plantago* leaf. **b** Leaves are split at the base of the leaf and the petiole, allowing for vascular tissue to be exposed. **c,d** Petiole tissue is pulled gently from the leaf, resulting in the isolation of the pure vascular tissue. **e** Pure vascular tissue and the empty petiole. **f** Extracted vascular tissue

Very few species have vascular tissue which is easily removed. The vascular tissue of celery is easily mechanically isolated from celery (Pommerrenig et al. 2006). While this extraction is simple, vascular tissue can only be extracted from the petiole, which can be a limitation for many studies. Additionally, celery is not an attractive model due to its complex breeding system and readily difficult to transform, necessitating an alternative model for vascular studies (Liu et al. 2022). In contrast, vascular tissue can easily be extracted from the leaf and petiole tissue of species in the *Plantago* genus (Fig. 3). This is due to the presence of a complete endodermal tissue that surrounds the vascular bundle, a unique anatomical feature to *Plantago* species. (Pommerrenig et al. 2006). This structural feature allows for the direct extraction of vascular tissue at high purity and yield, a method not commonly feasible in current plant model systems. An example of this extraction method is shown in Fig. 4. In contrast to conventional phloem sap collection techniques such as EDTA-facilitated exudation or stylectomy, which suffer from contamination or low yield, or cucurbit bleeding which is genus-specific, *Plantago* allows for the isolation of several grams of pure vascular tissue without requiring specialized equipment or transgenic lines. This method is also compatible with plants grown in natural or stress-inducing environments, making it valuable for functional genomics and stress biology research. Given these challenges posed by many of the collection methods, this tissue-based method offers a practical and scalable alternative- one that has contributed to Plantago being increasingly recognized as a promising model species for vascular biology (Huang et al. 2019; Xia et al. 2025; Levengood et al. 2024). As a result of this feature, several significant discoveries in the field of vascular biology have been made using *Plantago*, which are highlighted below.

Pommerrenig and colleagues identified 5900 expressed sequence tags from the phloem vasculature of *Plantago* (Pommerrenig et al. 2006). This effort enabled the localization of key genes in the “Yang Cycle”, an important pathway responsible for recycling MTA to Met, within the phloem tissue. Our group used *P. major* to investigate the genes that are differentially expressed in plant vasculature under phosphate deficiency. This study led to the discovery of previously unknown vascular tissue specific genes and pathways involved in low-phosphorus responses (Huang et al. 2019).

Genes related to carbohydrate transport in the phloem have also been identified in *Plantago*. The *P. major* sucrose transporter, *sucrose transporter 2 (PmSUC2)*, was localized to the vascular tissue of *P. major* (Gahrtz et al. 1994)*,* and its sequence has been used as a reference to identify homologous transporter genes in several other species (Weig and Komor 1996; Meyer et al. 2000; Aoki et al. 1999). Other sucrose transporters, *sucrose transporter 1 (PmSUC1)* (Gahrtz et al. 1996) and *sucrose transporter 3 (PmSUC3),* have also been localized to *Plantago* vasculature*. PmSUC3* is expressed in both source and sink tissue and may be involved in sugar sensing and unloading within the plant (Barth et al. 2003), while *PmSUC1* is believed to facilitate sucrose unloading to flowers (Gahrtz et al. 1996). In addition to the sucrose transporters, the sorbitol transporters, *polyol transporter 1* and *2* (*PmPLT1* and *PmPLT2),* have been found to be localized to phloem tissue and play a role in salinity stress resistance in *P. major* (Ramsperger-Gleixner et al. 2004).

Vascular-mediated systemic signaling plays a crucial role when plants face mineral and nutrient deficiencies (Mishra et al. 2024). For example, one study used *P. major* to investigate the biochemical and physiological effects of boron deficiency. The findings revealed that boron deficiency leads to the differential accumulation of various nutrients and phytohormones between leaf mesophyll and vascular tissue (Pommerrenig et al. 2019). In another study, the phloem exudates of *P. lanceolata* were used for targeted metabolic profiling to understand the interaction between the phytohormones jasmonic acid (JA) and salicycic acid (SA) during herbivore damage (Schweiger et al. 2014). The results demonstrated significant crosstalk between the JA and SA pathways.

In most higher plants, such as Arabidopsis, carbon is primarily transported throughout the phloem as sucrose. In contrast, *Plantago* species translocate both sucrose and sorbitol, a polyol, making them an excellent model for examining how polyol-translocating plants respond to stress (Zhang and Turgeon 2018; Ramsperger-Gleixner et al. 2004). We used CRISPR/Cas9 to demonstrate that the sucrose transporter function in *Plantago* is similar to those sucrose transporters in other apoplastic species as the knock-out of the gene led to typical phloem loading disrupted phenotype. However, unlike other apoplastic loading species, this manipulation in *Plantago* is not lethal, indicating the transport of sorbitol could compensate the reduced translocation of sucrose. Future work in using CRISPR/Cas9 to knock-out the expression of sorbitol transporter and the resulted phenotype will shed more light on the importance of the gene in carbohydrate phloem transport and salinity stress. For example, a study on *P. major* demonstrated that sorbitol accumulation in the vasculature enhances cold stress tolerance, with its protective effects significantly amplified by higher applications of potassium (Ho et al. 2020). Another study revealed that sorbitol is preferentially loaded into the phloem in response to salt stress, underscoring its critical role in helping *Plantago* adapt to saline conditions (Pommerrenig et al. 2007)*.*

While research in Plantago is still less extensive compared to traditional models like Arabidopsis, existing studies indicate that many of the findings in Plantago are conserved across diverse plant species. For examples, “Yang cycle” has been found to be located in the phloem of Arabidopsis and Plantago (Pommerrenig et al. 2006); the importance of phloem companion cells in transport sucrose and regulating low phosphorus responses have also been found to be conserved between the two species as well (Huang et al. 2019). The unique attributes of Plantago, such as the ability to transport sorbitol alongside sucrose, further enhance its appeal for studying plants with those specific traits.

## Floral symmetry

Floral symmetry is a key area of study when examining plant species in detail, as it plays a critical role in ensuring reproductive success and the transmission of desirable genetic traits to offspring (Ma et al. (2017)). The evolution of the flower hundreds of millions of years ago provided angiosperms with enhanced reproductive efficiency and reliability (Chanderbali et al. 2016). Angiosperms represent approximately ninety percent of plant biodiversity with a wide variety of floral characteristics, including differences in size, color, shape, scent, flowering time, number of parts, and symmetry.

The two main categories of floral symmetry are radial and bilateral. Radial symmetry, also referred to as polysymmetry or actinomorphy, is defined by rotational and reflection symmetry. In radial symmetry, specific floral organs, like petals or stamens, are identical in shape and size, and are evenly distributed around the receptacle. In comparison, bilateral symmetry, also referred to as monosymmetry or zygomorphy, is a homoplastic trait that is characterized by reflection along a single axis (Citerne et al. 2010). Flowers can also be asymmetrical, lacking any pane of reflection. This is a rarer form compared to actinomorphy and zygomorphy (Frohlich 2006).

These floral characteristics discussed are a result of evolution-based development since the late Jurassic era (Chanderbali et al. 2016). To fully understand these organisms, it is crucial to incorporate the evolutionary components of floral development over the past 200 million years into modern research. Evolutionary-developmental biology (Evo-Devo research) interconnect multiple scientific fields: evolutionary history and processes, biogeographic knowledge, population biology, physiology and anatomy, and molecular structural biology. Additionally, Evo-Devo research provides insights into the evolutionary tendencies of specific clades, enhancing our understanding of their adaptations and diversification (Frohlich 2006).

**Fig. 5:**
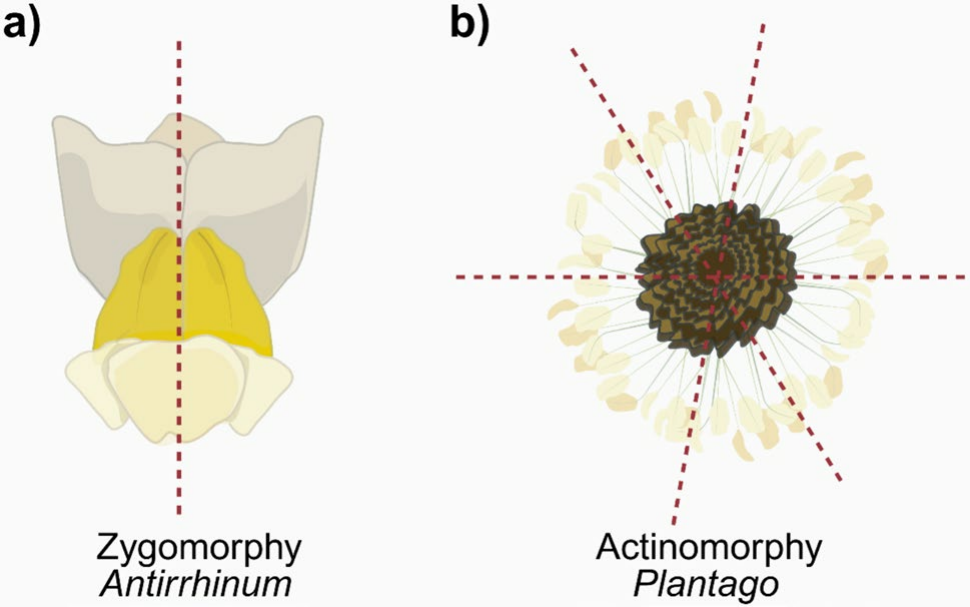
Although they belong to the same clade, floral morphology and symmetry differ between *Antirrhinum* and *Plantago.* **a** *Antirrhinum* flowers are zygomorphic (bilateral symmetry). **b** *Plantago* flowers are actinomorphic (radial symmetry)

Many species in the genus of *Plantago* have emerged as interesting systems for the Evo-Devo research, especially in the study of floral symmetry. Based on phylogenetic analyses, *Plantago* and *Antirrhinum* belong to the same clade within the Lamiales, commonly referred as the *Plantaginaceae* or the *Veronicaceae* (Olmstead et al. 2001). Interestingly, although *Plantago* and *Antirrhinum* are closely related, the flower structure and symmetries of these two genera are dramatically different—e.g. zygomorphy in *Antirrhinum* vs radial symmetry in *Plantag*o (Reeves and Olmstead 1998; Reardon et al. 2009). Figure 5 illustrates this floral divergence between *Antirrhinum* and *Plantago.* Although in the same clade, *Plantago* undergoes a genetic reversal to present actinomorphic flowers rather than zygomorphic (Frohlich 2006). The genetic diversity of the floral structure of *Plantago* provides an ideal system to study the evolution of flower development. Uses of *Plantago* for both Evo-devo and floral symmetry research are presented below.

The gene regulatory network controlling zygomorphy (bilateral symmetry) in *Antirrhinum* flowers has been well characterized (Luo et al. 1996). Within the regulatory loop, the *MYB* family gene *RADIALIS* (*RAD*) establishes the dorsal identity and confines the activity of another *MYB* gene *DIVARICATA* (*DIV*) to the ventral part of flowers. Through the dorsal–ventral regulation, zygomorphy can be established. On the other hand, although the inflorescences of the *Plantago* genus are polytelic and lateral, their flowers are radially symmetrical (actinomorphic), suggesting that any zygomorphic (bilaterally symmetrical) condition during early floral development in *Plantago* is likely ancestral (Reardon et al. 2009).

When previous studies were unable to identify either *DIV* or the *RAD* orthologs in *Plantago,* researchers hypothesized that these genes may have evolved novel functions in floral development, leading to the actinomorphic phenotype(Preston et al. 2011). In another study, the *DIV* ortholog was identified in *P. lanceolata* with high expression in inflorescence meristems and throughout all lateral floral organs(Reardon et al. 2014). The inability to detect RAD, coupled with persistent DIV expression in late-stage petal development is consistent with a RAD gene loss event(Tonsor 1985; Reardon et al. 2014). This supports the hypothesis that the evolutionary shift from zygomorphy to actinomorphy in *Plantago* was driven by the loss of dorsal gene function, resulting in an expanded domain of ventral gene expression and a consequent shift in floral symmetry.

This genetic reversal observed in *Plantago* offers a valuable model for understanding how gene loss can drive the evolution of ecologically significant traits, such as floral symmetry. The absence of the *RAD* gene in *Plantago* species allows for an expanded expression domain of ventral floral symmetry genes. This shift not only alters floral morphology but also contributes to inflorescence development, shedding light on the flexible roles of symmetry genes in closely related species and highlighting gene loss as a potential mechanism for morphological innovation.

Functional studies targeting the regulatory dynamics of *DIV* and *RAD* would be instrumental in testing the hypothesized genetic basis for actinomorphy in *Plantago.* Specifically, downregulation or targeted silencing of *DIV* in *Plantago* could clarify its functional role in the establishment of floral symmetry in the absence of RAD. Complementation experiments involving heterologous expression of *Antirrhinum RAD* in *Plantago* could reveal whether reintroduction of dorsal identity cues can re-establish zygomorphic features or modulate *DIV* expression. Recent advancements in *Plantago* as a model system, including the development of an efficient transformation system and the availability of sequenced genomes will enhance the feasibility of such experiments and allow for a more detailed exploration of symmetry evolution.

## Evo-devo patterns

*Plantago* species have also been used to investigate Evo-Devo patterns associated with environmental gradients, including those associated with temperature. *P. lanceolata* was used to investigate the evolutionary causes of plasticity in floral thermal reflectance. The results helped to confirm the hypothesis that floral thermal plasticity is adaptive to environments with shorter growing seasons, cooler temperatures, and higher altitudes (Marshall et al. 2019). Further, *P. lanceolata* was also used to study the interaction between prezygotic and postzygotic temperature with parental effects across generations in flowering phenology. The results indicated that the grandparental temperature (GPT) factor combined with the maternal family impacted flowering time and male sterility. For a species growing in varying temperature regions to evolve and display diverse breeding patterns, it will need to possess these genotype-specific responses (Case et al. 1996).

Another study used *P. asiatica* to investigate the evolutionary effects of exposure to high CO_2_ levels. Populations grown in areas with high CO_2_ presented heritable phenotypic and genetic differences from populations grown in areas with ambient CO_2_, indicating that evolutionary differentiation can occur in plant populations across various CO_2_ gradients (Nakamura et al. 2011). Similarly, in another study, *P. lanceolata* collected in the wild from 1905 until 2019 was used to evaluate the effects of climate change on plant phenology and morphology. A Co-inertia analysis showed that 34% of morphological traits, such as the length of petioles, leaf blades, and spikes, were associated with climate change. The same data presented that the temperature during plant bud development impacted spike length and higher temperatures during flowering impacted leaf length (Prokhorova and Netsvetov 2020).

These studies underscore the importance of considering evolutionary processes when examining the interplay between environmental factors and genetic responses in floral symmetry. The genetic reversal in *Plantago*, resulting in actinomorphic flowers and distinguishing it from closely related species, makes this genus a unique and valuable system for studying the evolutionary dynamics of floral development. Leveraging *Plantago* as a model species in future research promises to advance genetic analysis, linking genotypic responses to floral symmetry observed in both historical and contemporary contexts, and paving the way for deeper insights into the evolution of plant morphology.

## Male sterility and gynodioecy

Gynodioecy, a common reproductive system identified in many plant families, is characterized by the conjunction of female and hermaphrodite plants within a population, with hermaphrodites possessing both male and female reproductive organs. This system plays a crucial role in plant breeding and hybrid seed production for many agronomically important crops, promoting significant research interest in both academic and industry settings. Gynodiocey is common in plant families with flowers that contain a small, fixed number of ovules, such as *Plantago* in the *Plantaginaceae* family. The inheritance of male sterility among gynodioecious phenotypes is controlled by the interaction of nuclear and cytoplasmically inherited factors- specifically, cytoplasmic male-sterilizing genes and nuclear fertility restoring loci (Nugent et al. 2019). When male sterility is controlled by nuclear inherited genes, female plants must be twice as prolific as hermaphrodites for successful reproduction (Shykoff et al. 2003). In contrast, when male sterility is controlled by cytoplasmic factors, female plants can sustain and further generate a population even with limited fecundity (Lewis 1941). Most studied gynodioecious species exhibit sterility through cytoplasmically inherited genes (Shykoff et al. 2003). However, some species in the *Plantago* genus have been found to be the exception to this rule, with their sterility derived from both nuclear and cytoplasmic inheritance.

A few of the species in the *Plantago* genus display a typical gynodioecious phenotype, meaning that both hermaphrodite and female plants exist in the population, with the female plants having non-functional male organs. A few pioneer studies in eludicating the mechanisms underlying the sex determination and male sterility were conducted by a few botanists who used *Plantago* in their studies over one and a half centuries ago. For example, this phenomenon was first described in plants in 1876 using *P. lanceolata* as a model (Coleman 1876). It was discovered that male sterility in *P. lanceolata* is inherited through both nuclear and cytoplasmic inheritance (Lewis 1941), unlike other species where sterility is only inherited through the cytoplasm. Data obtained from studying female lines of several species of *Plantago* with male sterility led scientists to conclude that the male sterility types are associated with four distinct phenotypes (Gouyon et al. 1991), male sterile 1, 2, 3 and 4, which are associated with genes that have yet to be identified. It is thought that each of these phenotypes are caused by a mitochondrial cytoplasmic factor CMS, of which four distinct types have been identified for *P. lanceolata* (De Haan et al. 1997). Physiological studies on *P. lanceolata* flowers indicated that cytokinin could be involved in determining the differences between the sex types in the species (Olff et al. 1989). Most recent studies in this field hypothesize that male sterility *in P. lanceolata* is caused by a signal that triggers cell death, which is initially perceived by epidermal cells and then is translocated inward across anther cell walls (Nugent et al. 2019).

While CMS has been well-characterized in many cultivated crop species, these species have the advantage of being extensively bred and domesticated, which may result in sterility mechanisms that do not fully reflect the diversity seen in wild populations (Van Damme 1983). Since CMS is commonly used in hybrid crop development, studying its mechanisms in wild populations exhibiting this phenomenon is essential for adapting new plants for crop production. *P. lanceolata* offers a unique model for studying CMS, as its sterility mechanism differs from conventional models, potentially providing a more accurate representation of how CMS functions in wild plant populations. Additionally, some *Plantago* species do not display male sterility, suggesting that flower development regulation can vary significantly within the same genus. Comparative genomics and transcriptomic studies between *P. lanceolata* and non-CMS Plantago species could uncover genes and pathways associated with male sterility. Recent advancements in Plantago molecular tools, such as transformation techniques, will further facilitate these investigations.

## Abiotic stress

Due to their sedentary nature, plants are significantly affected by abiotic environmental stress, which often represents the largest impact on their growth and development (Zhang et al. 2023). Because these stresses greatly limit crop yields, understanding the mechanisms behind how plants respond and adapt to adverse conditions is critical for increasing global food security. Numerous studies have investigated the genetic, biochemical and molecular mechanisms in *Plantago* due to their adaptability to diverse environmental conditions. For example, two species of *Plantago, P. lanceolata* and *P. coronopus,* from contrasting environments—low altitude/high temperature and high altitude/low temperature—were examined for their seed traits adapted to these conditions. The study found that the two species exhibited different strategies to adapt to their environments, including efficient seed germination, offering valuable insights for conservation and restoration efforts (Teixeira et al. 2020a, b). Among these, *P. lanceolata* has demonstrated remarkable adaptability across various environmental conditions and has thus been used in many studies on abiotic stress. Another group studied *P. lanceolata* populations at various levels of combined drought stress and elevation to examine the mechanisms behind the adaptability to each condition. The results showed that *P. lanceolata* can adapt to high elevations by activating photo and antioxidant protection mechanisms, adjusting stress-related phytohormones and can regenerate its aboveground biomass after the stress has passed (Morales et al. 2021).

Drought stress has also been studied in various *Plantago* species, revealing unique physiological responses. In *P. lanceolata,* drought conditions were found to reduce foliar nitrogen compounds and the iridoid glycoside catalpol, while increasing the accumulation of sorbitol, malate, citrate and acucubin (Orians et al. 2019). Surprisingly, proline accumulation, which is commonly observed in other species under drought stress, was reduced in *P. lanceolata*. Another study examining root and shoot traits in *P. lanceolata* found that drought decreased the labile litter fraction in shoots of the plant, contrasting with the typical increase observed in other species (Reinelt et al. 2023). These findings underscore the unique adaptations of *Plantago* species to drought conditions.

Several *Plantago* species exhibit varying levels of salt tolerance, making them excellent models for comparative studies on responses to salt stress. Studies revealed that the salt sensitive *P. media* and the salt-tolerant *P. maritima* employ different strategies for ion uptake and distribution (Erdei and Kuiper 1979; Königshofer 1983). Electrophysicological analysis further indicated than an unknown mechanism regulating plant membrane channels under salt stress is differentially involved in these two species (Maathuis and Prins 1990). Additionally, *P. maritima* exhibits higher induction of antioxidant enzymes in response to salt stress compared to *P. media* (Sekmen et al. 2007). Analysis of Na + , Cl‾, K + , and proline levels in salt-treated *Plantago* species implies that a high efficiency in the transport of toxic ions to the leaves, the capacity to use inorganic ions as osmotic compounds, as well as proline accumulation and K + transport to the leaves are some of the potential mechanisms enabling tolerance in the genus *Plantago* (Al Hassan et al. 2016). In another study, the expression of two phloem expressed genes—a *sorbitol transporter* and a *sucrose transporter—*showed opposite responses to salinity (Pommerrenig et al. 2007). The expression of sorbitol transporter genes, *PmPLT1* and *PmPLT2,* are rapidly up-regulated in response to salt treatment, while the mRNA levels of the sucrose transporter gene, *PmSUC2,* is downregulated. This demonstrates that *Plantago* is capable of adapting its carbon partitioning to varying environmental conditions by upregulating the expression of sorbitol transporter genes. It was speculated that the increased activity of sorbitol export, driven by higher expression of the *sorbitol transporter*, is correlated to the adaptive responses in *P. major* to salt. However, a definite function of sorbitol transport in relation to salinity stress cannot be confirmed unless the manipulation of the gene in Plantago and the resulted phenotype are performed.

## Phytoremediation

Pollutants, particularly pesticides and heavy metals, remain a major threat to soils and aquatic environments worldwide. Pesticides accumulate in soils as the result of agricultural application. Heavy metals are environmental pollutants resulting from industrial activities and persist in ecosystems due to their inability to naturally degrade. Existing physical and chemical methods to remove these pollutants from soil and water are expensive, limiting their application (Aioub et al. 2019). In contrast, phytoremediation is one of the most environmentally friendly and cost-effective approaches for cleaning contaminated soils. This environmentally friendly approach utilizes plants with the ability to accumulate or break down pollutants, known as bioaccumulators, and has proven highly effective in mitigating soil and water pollution worldwide. Many *Plantago* species have been proven to be excellent bioaccumulators and bioindicators of heavy metals in soil. *P. lanceolata* and *P. tunetana* have demonstrated tolerance to zinc, copper and iron during germination, accumulating these metals without significant toxic effects (Ltaeif et al. 2024) (Andreazza et al. 2015). Additionally, *P. major* was found to be effective in reducing lead (Romeh et al. 2015) and nickel (Lyu et al. 2024), while *P. lagopus* effectively mitigates aluminum toxicity in soils (Correia et al. 2014).

*P. major* has demonstrated significant potential in reducing several pesticides in water and soil, including the insecticides cypermethrin (Aioub et al. 2021) and Imidacloprid (Romeh 2009), the pesticide cyanophos (Romeh 2014) and the fungicide azoxystrobin. The agricultural application of *P. major* as a bioaccumulator was highlighted in an intercropping study with tomatoes in cypermethrin contaminated soils. Intercropping effectively reduced cypermethrin levels and protected the tomato crops from any harmful effects of the insecticide. *P. media* has also been effectively used for pesticide phytoremediation but is considered to be underutilized, and thus should be explored for further research potential (Fierascu et al. 2021).

Several genes and pathways contributing to heavy metal tolerance have been discovered in *Plantago* species. For instance, researchers found that a type 2 *Metallothionein* (*PoMT2*) gene could be involved in the overaccumulation of heavy metals in *P. ovata* because there is a correlation between the ZnSO_4_ induced *PoMT2* expression and the increased antioxidant activity and α,α-diphenyl-β-picrylhydrazyl (DPPH) radical scavenging activity (Pramanick et al. 2017). Although promising, the pathways require further molecular validation before a definite role is assigned to these genes. In *P. major,* differentially expressed genes related to nickel tolerance have been identified*.* Pathways related to growth and photosynthesis were significantly reduced, pathways related to metabolite synthesis, chitin synthesis and adversity signal transduction were stimulated, and pathways related to root cell wall organization or biogenesis were suppressed. Notably, the rate limiting enzyme, PmHISN1A/B, in the histidine synthesis pathway was shown to play a role in nickel tolerance (Lyu et al. 2024). Transgenic Arabidopsis plants with increased expression of this gene led to enhanced Ni tolerance.

As demonstrated above, *Plantago* species have shown significant potential in identifying some of the genes that are related to heavy metal tolerance. In the case of *PmHISN1A/B*, further functional characterization-including the analysis of differentially expressed genes and pathways under heavy metal stress, as well as heterologous expression of the gene in another plant species- conformed its role in enhancing heavy metal toxicity resistance. A similar approach could be applied to discover and validate the function of other *Plantago* genes, like *PoMT2* in heavy metal stress resistance. The application of these genes holds promise not only for improving crop resistance to heavy metal contamination but also for expanding the range of plant species that can be used as bioaccumulators in phytoremediation efforts.

The broad applicability of several *Plantago* species as bioaccumulators makes them ideal for phytoremediation. Their ability to breakdown various pesticides and to accumulate and tolerate heavy metals makes them valuable models for developing strategies to mitigate soil contamination. Current studies have already demonstrated such application of these findings, including intercropping to protect economically important crops from pesticides in contaminated soils. Additionally, the identification of key genes and pathways involved in heavy metal tolerance further enhances the potential of *Plantago* as a model for developing more resilient crops. Continued research into these species and their genetic mechanisms will likely lead to more effective and sustainable approaches to environmental conservation.

## Plant–microbe interactions

Plant–microbe interactions are a vital part of the evolution of plant biodiversity (Igwe et al. 2021). Several species in the genus *Plantago* have been used to unlock the mechanisms behind plant microbe interaction*. Plantago* as a weed in the wild are hosts for many plant viruses, making them valuable models for investigating the pathology of infections.

Several *Plantago* species have been used to investigate the pathology and consequences of plant-pathogen interactions, particularly with downy mildew disease, which is caused by the pathogen *Peronospora plantaginis*. One study investigated the mechanisms of the disease in large scale *P. lanceolata* populations. Results revealed that overwintering conditions play a critical role in the pathogen population dynamics, with mild winter causing an increase in disease prevalence in the host plant populations (Penczykowski et al. 2015). In another study, *P. ovata* was used to identify the consequences of downy mildew disease on plant photosynthesis. It was found that the disease decreases photosynthesis rates by reducing chlorophyll content and causing an increase in starch accumulation in infected leaves (Mandal et al. 2009).

The perennial nature of some species in the *Plantago* genus often allows for viruses to persist in the plants throughout cold environments, making them valuable for investigating the pathology and consequences of infection. RNA-seq was used to identify four novel viruses, belonging to the *Vaulimovirus, Betapartitivirus, Enamovirus,* and *Closterovirus* genera, hosted by *P. lanceolata* (Susi et al. 2019). This study highlighted the diversity of the plant viral community, revealing evidence of viral infection in populations regardless of the presence of obvious symptoms, thereby opening up opportunities for further research. Plantago asiatica mosaic virus, initially identified in *Plantago asiatica*, has facilitated diverse research efforts aimed at understanding the virus and developing strategies to mitigate infection (Komatsu and Hammond 2022).

In addition to their use in studying plant-pathogen interactions, *Plantago* species have been employed to investigate the effects of beneficial soil microbes on plants. For example, the bacterium *Curtobacterium flaccumfaciens* strain E108, isolated from the rhizosphere of the salt-tolerant *P. winteri*, was shown to enhance salt stress tolerance in barley. Inoculation with this bacterium increased barley growth by up to 300% under high-salt conditions (Cardinale et al. 2015). A similar strategy was applied to *P. major* to increase its effectiveness in phytoremediation. In this study, *P. major* was inoculated with EM1, a widely used plant probiotic, to assess its potential as a host. The inoculated plants exhibited enhanced phytoremediation capacity, successfully remediating soil contaminated with the pesticide imidacloprid (Romeh et al. 2015). These findings confirmed the potential of *Plantago* as a host for EM1 and showed that their interaction may be important to increasing future phytoremediation strategies.

Pathology and plant–microbe interactions are deeply interconnected throughout a plant’s lifecycle. Understanding these interactions is essential to uncovering how nutrients and pathogens influence plant health. For example, *Plantago* is often used as a host plant to model the effects of arbuscular mycorrhizal (AM) fungi interaction with plants. Several reports have shown that AM fungi are capable of increasing the bioaccumulation efforts of *Plantago,* including for heavy metals such as arsenic (Orłowska et al. 2012) and chromium (Nogales et al. 2012), indicating that AM fungi inoculated *Plantago* could be useful for the bioremediation efforts of contaminated soils.

Beyond nutrient uptake, the role of AM fungi in disease resistance has also been investigated in *Plantago.* Research indicates that these fungi have an indirect benefit in improving plant defenses against pathogens (Fontana et al. 2009)and have an effect in defense against herbivores (Gange et al. 2002), although the exact mechanisms that mediate these benefits have yet to be studied. The AM fungi-*Plantago* system has also been proven useful in examining how environmental factors influence plant-pathogen dynamics. While there are no reports on how soil pH impacts microbes using *Plantago,* one study used *P. lanceolata* to investigate the effects of soil moisture and temperature on AM fungal colonization, using soil sourced from various climates. The study found that while soil temperature and moisture have a positive impact on plant growth, only higher temperatures had a similar impact on AM colonization rates (Rasmussen et al. 2020). Although studies into how other environmental factors impact AM fungi is limited in *Plantago,* this study shows that *Plantago* is an effective host system for future efforts.

While these studies show that *Plantago* species are ideal models for modeling the plant-pathogen interaction effects of pathogens like AM fungi, many of these studies lack functional characterization of the biochemical or genetic pathways that mediate these interactions, meaning that future efforts have to be made before these discoveries can be applied to other species. For example, vascular tissues are known to mediate the signal transduction between shoots and roots/other sink tissues to certain diseases. Genes and pathways identified from the vascular tissue of *Plantago* infected by both beneficial microbes like AM fungi or pathogens that mediate disease can then be translated to other crop species.

## Secondary compounds and medicinal chemistry

In the United States, species of the *Plantago* genus are generally regarded as weeds or forage plants, with their traditional medicinal and nutritional values largely unrecognized. In contrast, in many other parts of the world, particularly in Asia, *Plantago* has been widely used in both traditional and modern medicine.

For example, it has been confirmed that *P. major* is a viable treatment for medical conditions such as asthma, urinary disorders, and dermatologic issues, and it has been recommended as a valuable source for the manufacturing of drug products (Najafian et al. 2018). Many species of *Plantago,* including *P. major* and *P. ovata,* are myxospermous, meaning that they release mucilage from their seeds. In *P. ovata,* dried mucilage can be removed from the seed mechanically and is called psyllium. This product can be used as a dietary supplement to improve intestinal health (Gonçalves and Romano 2016). The seed husks can also be mixed with water to treat hyperglycaemia in both type 1 and type 2 diabetes (Hannan et al. 2006). Additionally, the gel-like polysaccharides extracted from *Plantago* seeds are used for toxin removal and as a medium for drug delivery (Gonçalves and Romano 2016). *Plantago* products are also used to treat upper respiratory tract diseases, as well as mouth, throat, and skin conditions (Oloumi et al. 2011).

In addition to their established medicinal applications, myxospermous species such as those within the *Plantago* genus have become important models for studying cell wall formation (Phan et al. 2020). While Arabidopsis has traditionally served as a model organism in this field, its small seed size and relatively low mucilage yield present limitations for detailed genetic, biochemical and functional analysis. In contrast, several *Plantago* species, especially *P. ovata,* produce larger seeds with significantly higher mucilage output, making them more suitable for such large investigations. In a recent review, Cowley and Burton (2021) have comprehensively summarized the findings related to cell wall formation, including mucilage biosynthesis and seed coat development in *P. ovata* and related species and will therefore not be discussed in detail here. Notably, the authors also highlight *Plantago* as an emerging model organism in this field, underscoring its growing relevance across multiple areas of research.

The biochemistry behind these confirmed health benefits of *Plantago* is also a critical point of investigation in medicinal studies. Several bioactive compounds have been isolated and characterized from nearly all parts of the plant, including flavonoids, alkaloids, terpenoids, phenolic acid derivatives, iridoid glycosides, fatty acids, polysaccharides and vitamins (Nishibe et al. 1995; Beara et al. 2009; Handjieva et al. 1991). Extracts from *P. major* have traditionally been used as a wound healer and for their anti-ulcerative, antidiabetic, antidiarrhoeal, anti-inflammatory, anti-nociceptive, antibacterial, antiviral agent, antioxidant, and free radical scavenging properties (Adom et al. 2017; Zubair et al. 2016). While the bioactive compounds in *Plantago* have been identified, the mechanisms responsible for the biosynthesis of them are largely unknown (Gonçalves and Romano 2016). Identifying these pathways has been historically challenging, due to limited genomic resources that exist in *Plantago,* such as limited species in the genus having sequenced genomes and few functional genomics tools like plant transformation and gene editing. With improved sequencing resources, and a robust transformation system (described in the later part), researchers will be able to uncover the genes and pathways related to the biosynthesis of these important natural compounds. Future genetic engineering efforts on the discovered genes can improve their production in these plants.

## Other ecological findings

With their widespread geographical distribution, plasticity to adapt to various environmental conditions, and unique interactions with other organisms, species in the *Plantago* genus have also been proposed as excellent models for ecological studies (Penczykowski and Sieg 2021).

Herbivory interactions, which examine how plants interact with animals and other organisms, represent a key area of ecological research. In one study, a group of researchers found that a population of the butterfly *Euphydryas Editha,* which originally laid eggs on the native plant *Collinsia parviflora,* gradually evolved a preference for laying eggs on *P. lanceolata*. *P. lanceolata* plants are non-native and were introduced to the habitat by cattle ranchers. However years later, when cattle ranching ceased and the land became dominated by other plant species rather than *P. lanceolata*, these butterflies went to extinction, despite the abundant availability of their original native host plants. This discovery laid the foundation for a new theory known as the eco-evolutionary trap (Singer and Parmesan 2018). In addition to ecological research, several studies have examined the dietary effects of *Plantago* on various agricultural livestock, and found that the introduction of *Plantago* to animal diets have beneficial effects to the animals. *P. lanceolata* is known to have a diuretic effect on pastoral dairy cattle (Al-Marashdeh et al. 2021), and integrating it into cattle diets can reduce nitrous oxide emissions, a major contributor to greenhouse gases originating from cattle urine (Simon et al. 2019). Interestingly, several animals, including farmed deer, lamb and sheep, exhibit a preference for *P. lanceolata* when given a choice.

A few studies have also demonstrated that *Plantago* can serve as valuable systems to study gene flow and evolution (Tonsor 1985; Bos et al. 1986). For example, a comprehensive study, in which the mitochondrial gene *atp1* in 43 *Plantago* species were analyzed using molecular and phylogenetic approaches, discovered novel instances of the horizontal gene transfer from the parasitic flowering plants to the host *Plantago* plants (Mower et al. 2004). This discovery has significantly expanded our understanding of gene flow and the complex interactions between parasitic plants and their hosts.

## Plantago species as new plant model organisms

Despite their versability in various areas of research, *Plantago* species cannot fully realize their potential as model organisms based on their research applications alone. As science and technology advances, research organisms require advanced genetic tools, such as a genetic manipulation system and a fully sequenced genome, to effectively answer complex research questions and address the relationship between the genome and phenome. In recent years, there has been growing interest in developing such tools for *Plantago* species, and additional tools are expected to emerge as more scientists recognize the research potential of *Plantago.*

## Sequencing

Sequencing technologies have been employed to investigate the genomes of various *Plantago* species. The largest amount of sequencing data exists for *P. major,* which is unsurprising, given its status as one of the most studied species in the genus. Nanopore and Hi-C sequencing technologies were used in conjunction to develop a genome-level assembly of *P. major,* and 31,654 protein-coding genes were identified in the genome (Lyu et al. 2023). Additionally, 5900 expressed sequence tags from *P. major* vascular tissue have been sequenced (Pommerrenig et al. 2006). The chloroplast genomes for multiple species in the genus have also been sequenced, including *P. major* (Liu et al. 2024a, b)*.* The full genome level assembly has also been published for *P. ovata*, identifying 41,820 protein-coding genes, 411 non-coding RNAs, 108 ribosomal RNAs, and 1295 transfer RNAs (Herliana et al. 2023). The detailed summary of the sequenced *Plantago* genomes is listed in Table 2.

**Table 2:** Sequencing of *Plantago* species

*Plantago* species	Level of assembly	Citations
*P. major*	Chromosome level assembly Vascular-specific EST Chloroplast genome	Lyu et al. (2023) Pommerrenig et al. (2006) Liu et al. (2024a, b)
*P. asiatica*	Chloroplast genome	Si et al.(2022)
*P. media*	Chloroplast genome	Min and Tao (2020)
*P. lanceolata*	Chloroplast genome	Mehmood et al. (2024)
*P. argentea*	Chloroplast genome	Mehmood et al. (2024)
*P. atrata*	Chloroplast genome	Mehmood et al. (2024)
*P. maritima*	Chloroplast genome	Mehmood et al. (2024)
*P. ovata*	Chromosome level assembly	Herliana et al. (2023)

## Plant transformation systems

**Fig. 6:**
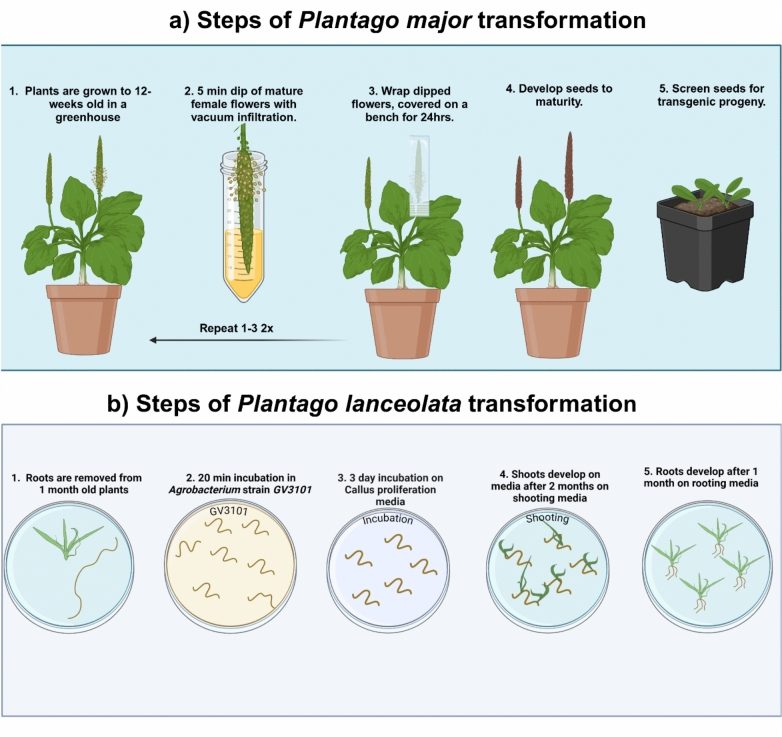
*Agrobacterium tumefaciens*-mediated transformation methods in *Plantago.* **a** *P. major* floral dip transformation, adapted from Pommerrenig et al. (2006) **b** *P. lanceolata* tissue-culture based transformation system, adapted from Levengood et al. (2023) This image was generated using biorender.com

Establishing an efficient plant transformation system is a crucial step in advancing a species as a model organism. *Agrobacterium tumefaciens*-mediated transformation is the most widely used plant transformation technique due to its broad applicability across plant species. For *P. major*, a system was developed by modifying the widely adopted Arabidopsis floral dip technique (Pommerrenig et al. 2006). Additionally, a tissue-culture based transformation system has been developed for *P. lanceolata* (Levengood et al. 2023). Using root tissues of 3–4 week-old plants as explants, transgenic plants could be obtained at a transformation efficiency of approximately 20%. A summary of *Agrobacterium tumefaciens*-mediated transformation methods is illustrated in Fig. 6.

Hairy root-based transformation systems have also been developed for *P. major* and *P. lanceolata*. While hairy root transformation only induces transgenic roots, potentially limiting its applicability for certain studies, it remains a highly valuable tool for research. *P. major* hairy root cultures were obtained with *A. rhizogenes* strain A4 and modified culture media (Rahamouz-Haghighi et al. 2023). The same bacterial strain was also used to induce transgenic hairy roots in *P. lanceolata* using leaf explants (Rahamouz-Haghighi et al. 2021).

The establishment of transformation systems for *P. major* and *P. lanceolata* represents significant progress in advancing *Plantago* species as valuable research models. Expanding transformation systems to additional species within the genus, such as *P. maritima*, would further enhance opportunities for molecular and physiological studies. For example, *P. maritima*’s remarkable salt tolerance could be studied in greater detail, potentially informing applications in other species. Such advancements will likely continue to progress as more research groups adopt *Plantago* as applicable research organisms.

## Gene editing

The innovation of CRISPR-Cas9 gene editing has allowed for scientists to perform more precise experiments that explore the interaction between the genome and phenome of plants. For emerging model species like *Plantago,* it becomes essential to evaluate the compatibility of CRISPR with these new systems.

Until recently, the application of CRISPR-Cas9 in *Plantago* species faced significant challenges, primarily due to the lack of sequenced genomes and reliable transformation systems. Since sequencing information is necessary to both design the guide RNAs and mitigate off-target effects, these limitations made it difficult to apply gene editing to *Plantago.* Despite these challenges, there have been notable advancements in the indirect application of CRISPR technology to *Plantago* species. For example, seed cell wall genes GT61 and DUF579 from *P. ovata* were edited in Arabidopsis protoplasts (Herliana 2017).This highlights a growing research interest in applying gene editing strategies to species in the *Plantago* genus.

**Fig. 7:**
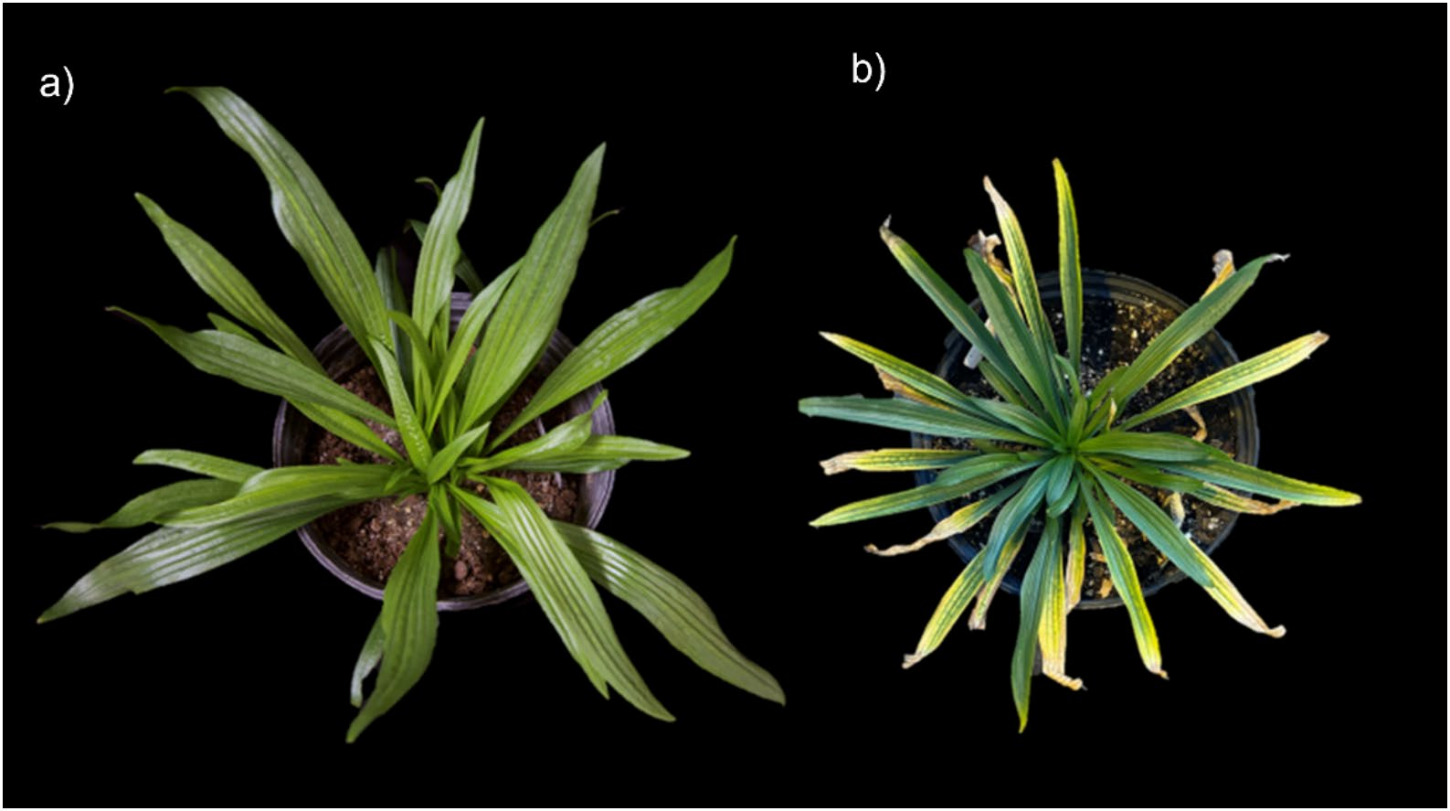
CRISPR can be applied to *Plantago lanceolate—*A phloem-localized sucrose transporter gene was successfully knocked out in *P. lanceolata,* using CRISPR-Cas9. **a** 2-month old WT *Plantago.* **b** 10 month old edited plant. The retarded growth and chlorotic pattern of the edited plants display classical symptoms of phloem loading inhibition, confirming CRISPR compatibility in this species

Using our recently developed transformation system, our group has successfully knocked out the sucrose transporter gene in *P. lanceolata* using CRISPR-Cas9. The edited plants exhibited chlorotic leaves and retarded growth, typical phenotypes associated with disrupted sucrose phloem loading, demonstrating the effective application of CRISPR in *P. lanceolata* (Fig. 7).

## Conclusions and future perspectives

As highlighted in this review, *Plantago* species have been widely used to study vascular biology, stress physiology, ecology and evolution, medicinal chemistry, cell wall formation, and floral development (Gonçalves and Romano 2016; Najafian et al. 2018; Penczykowski and Sieg 2021; Huang et al. 2019; Levengood et al. 2023, 2024; Pommerrenig et al. 2006). Additionally, several underutilized species within the genus have been recognized for their future research potential. For instance, *P. media* holds promise for applications in both medicine and nanotechnology (Fierascu et al. 2021), while *P. rugelii* offers valuable opportunities for studying environmental gradients on a regional scale. These examples emphasize the broad and expanding application of *Plantago* in diverse areas of scientific research.

Until recent years, the adaptation of *Plantago* species as model organisms has been limited due to the lack of biotechnological tools and sequenced genomes. However, significant advancements over the past 5 years have greatly increased the potential of *Plantago* for scientific research. Genetic transformation systems have been developed for both *P. lanceolata* and *P. major,* and the fully sequenced genomes of *P. ovata* and *P. major,* along with multiple chloroplast assemblies will enable more in-depth analyses, particularly in linking the genome and phenome. The successful application of *Agrobacterium*-mediated transformation methods with CRISPR-Cas9 in *P. lanceolata* opens new avenues for functional genomics studies of genes and pathways. For example, the genetic mechanisms behind several unique features of *Plantago,* like gynodieocity in *P. lanceolata,* salt-resistance of *P. maritima,* and the phytoremediation capacity of *P. major* could be unlocked. These insights could then be applied to improve the resilience of crop species. In addition, understanding the genetics behind the curious wound-healing properties of *Plantago* species, especially psyllium-producing species like *P. major* and *P. ovata*, holds the potential for advancing human medicine. As the research potential of *Plantago* continues to be recognized, the development of biotechnological tools is expected to expand to additional species within the genus, further enhancing its utility in diverse fields of study.

Beyond their role as model species in basic research, *Plantago* species hold significant potential for applications in pharmaceuticals, cosmetics, and various health-related products. For example, identifying and overexpressing the genes responsible for psyllium production in *Plantago* could enhance psyllium yields. This advancement would be particularly valuable for drug manufacturers, who frequently use psyllium extracts as pharmaceutical binding agents. Such applications demonstrate the practical and commercial importance of *Plantago* species, extending far beyond their academic research potential.

Given the breadth of research conducted on *Plantago* and recent advancements in plant biotechnological tools, *Plantago* species are well-positioned to study gene functions underlying certain biological processes that cannot be effectively addressed using other model plants.

## Data Availability

No datasets were generated or used for this review paper.
